# Tumor models in various *Drosophila* tissues

**DOI:** 10.1002/wsbm.1525

**Published:** 2021-03-21

**Authors:** Shangyu Gong, Yichi Zhang, Aiguo Tian, Wu-Min Deng

**Affiliations:** Department of Biochemistry and Molecular Biology, Tulane University School of Medicine, New Orleans, Louisiana, USA

**Keywords:** cachexia, cancer, *Drosophila*, stem cell, tumor

## Abstract

The development of cancer is a complex multistage process. Over the past few decades, the model organism *Drosophila melanogaster* has been crucial in identifying cancer-related genes and pathways and elucidating mechanisms underlying growth regulation in development. Investigations using *Drosophila* has yielded new insights into the molecular mechanisms involved in tumor initiation and progression. In this review, we describe various tumor models that have been developed in recent years using different *Drosophila* tissues, such as the imaginal tissue, the neural tissue, the gut, the ovary, and hematopoietic cells. We discuss underlying genetic alterations, cancer-like characteristics, as well as similarities and key differences among these models. We also discuss how disruptions in stem cell division and differentiation result in tumor formation in diverse tissues, and highlight new concepts developed using the fly model to understand context-dependent tumorigenesis. We further discuss the progress made in *Drosophila* to explore tumor—host interactions that involve the innate immune response to tumor growth and the cachexia wasting phenotype.

This article is categorized under:

Cancer > Genetics/Genomics/Epigenetics
Cancer > Stem Cells and Development
Cancer > Molecular and Cellular Physiology

## 1 | INTRODUCTION

*Drosophila melanogaster*, a model organism that plays an illustrious role in many branches of biological research, has contributed greatly to our understanding of complex pathological processes, including neurodegeneration, obesity, diabetes, and aging related diseases (Baker & Thummel, 2007; Demontis et al., 2013; Hirth, 2010). In cancer research, the *Drosophila* model has been crucial to the discovery of genes and pathways that play oncogenic or tumor suppressor roles, thanks to their shared functions in the development of multicellular organisms (Brumby & Richardson, 2005; Parvy et al., 2018; Sonoshita & Cagan, 2017; Stratton, 2011). One such gene is *lethal (2) giant larvae (lgl)*, first discovered in the *Drosophila* model (Gateff, 1978), and later found to be part of a group of cell-polarity genes that also include *scribble (scrib)* and *discs large (dlg)*. Mutations of these genes, collectively known as neoplastic tumor suppressor genes, lead to loss of apicobasal polarity in epithelial cells and neoplastic overgrowth in the larval brain and imaginal discs (Bilder et al., 2000a; Humbert et al., 2008). The human *scrib* homolog was later found to assume a tumor suppressor role in breast, liver, skin, and lung cancers (Elsum et al., 2012; Moreno-Bueno et al., 2008), highlighting the evolutionarily conserved function of this class of genes in tumor suppression.

**FIGURE 1:**
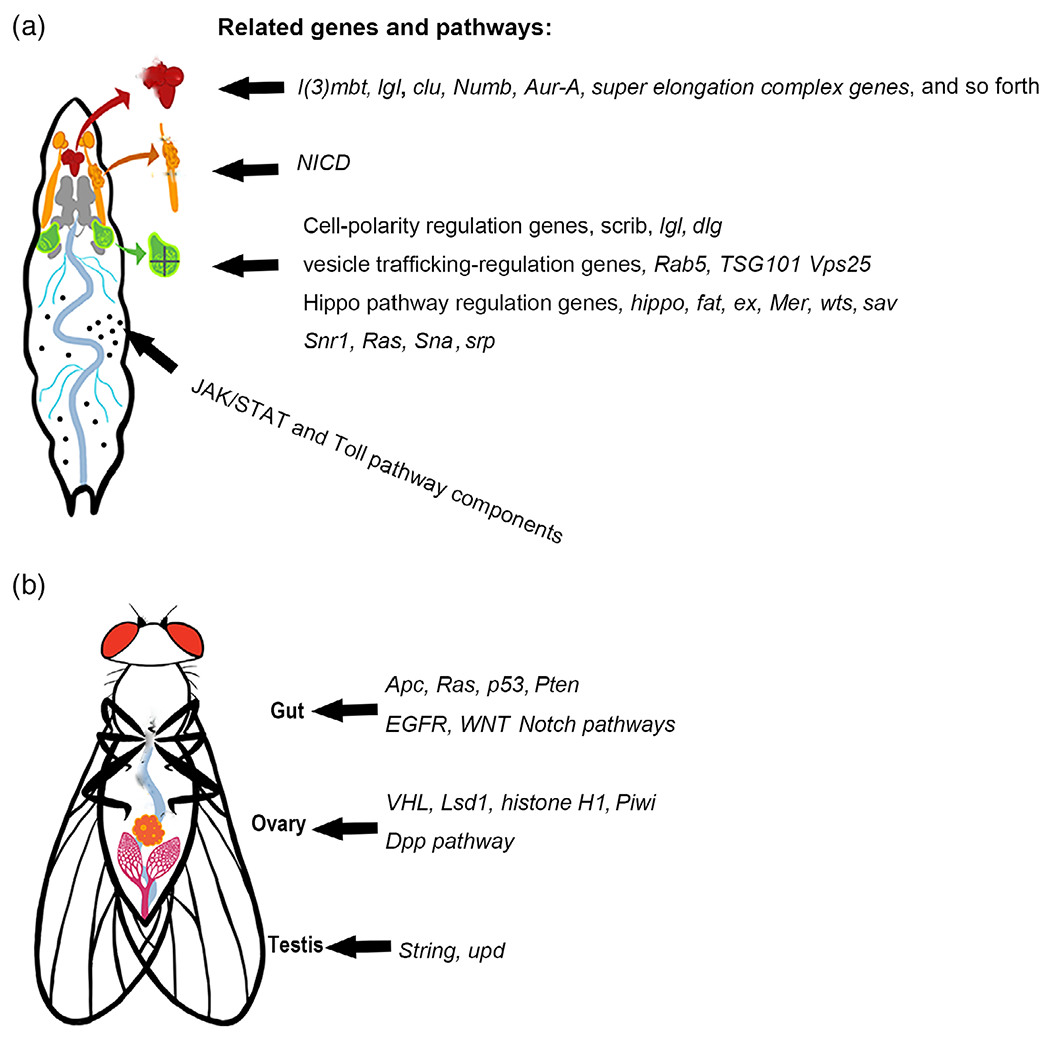
Tumor models and related genes and pathways in different larval and adult tissues. (a) Larval tumor models in the brain, salivary gland imaginal ring, imaginal discs and hemocytes. (b) Adult tumor models in the gut, ovary, and testis

**FIGURE 2:**
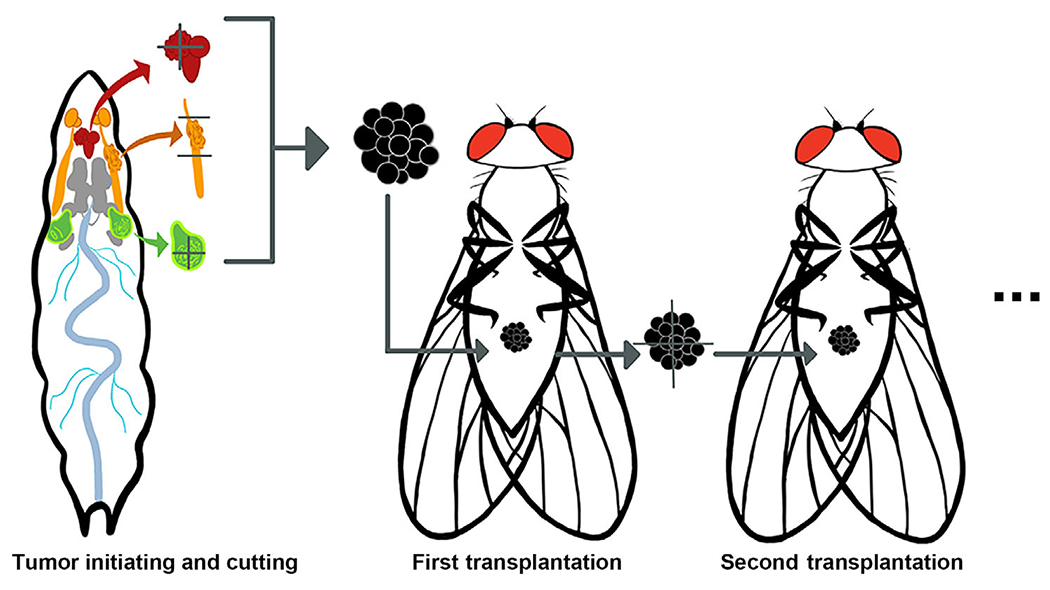
Tumor transplantation (xenograt) studies in *Drosophila.* First, primary tumors are induced in various tissues. They are then harvested and sectioned into small pieces. Next, the tumor pieces are injected into the abdomen of a host adult fly. The transplanted tumor can be harvested and replanted into new host flies for continued tumor growth

**FIGURE 3:**
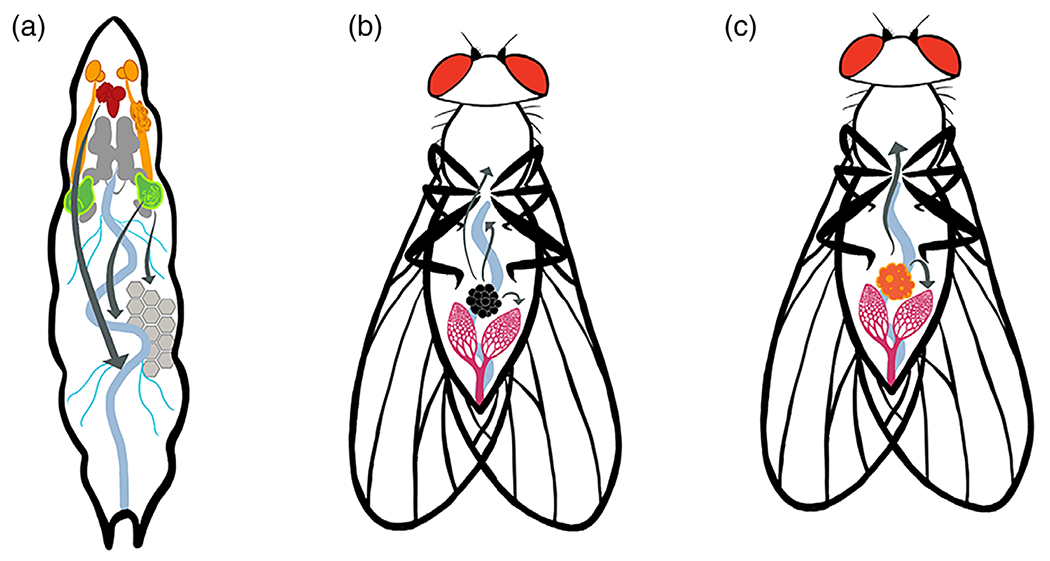
Metastasis in *Drosophila* tumor models. (a) The larval tumor cells metastasize to the gut, fat body, and trachea. (b) Tumor cells from the transplanted tumor metastasize to the gut, ovary, muscle, and brain. (c) The ISC tumor cells induced in the adult gut metastasize to the ovary and other tissues. (Arrows indicate metastasis directions)

In recent years, owing to the large arsenal of powerful and versatile tools available for genetic manipulation, a growing number of tumor models have been established in various *Drosophila* tissues to address fundamental questions of cancer biology. Among these tools, the flippase recombinase (FLP)/flippase recognition targets (FRT) based mitotic recombination technique, which can generate mosaics that induce tumorigenic mutations in clones, is particularly useful, as it resembles the process of cancer initiation (Potter et al., 2000). When this technique is combined with other tools such as the Gal4/Upstream Activation Sequence (UAS), RNA interference (RNAi), and Clustered Regularly Interspaced Short Palindromic Repeats (CRISPR)/Cas9 techniques, cancer-related genes can be manipulated in a strict spatial- and temporal-pattern for exploring mechanisms in tumor initiation, growth, metastasis, and cell—cell interactions within a tumor microenvironment (Chatterjee & Deng, 2019; Gonzalez, 2013; Herranz et al., 2016; Johnson & Halder, 2014; Sonoshita & Cagan, 2017; Stefanatos & Vidal, 2011). In this review, we focus on tumor models in various *Drosophila* tissues with human cancer hallmarks (Figure 1) (Hanahan & Weinberg, 2011), and explore consequences of tumor growth, including cachexia-like wasting and tumor—host interactions. Specific attention will be given to tumor models that are capable of transplanted growth and metastasis (Figures 2 and 3).

## 2 | TUMOR MODELS IN DIFFERENT TISSUES

### 2.1 | The imaginal tissue

#### 2.1.1 | Imaginal discs

The imaginal discs, which are structured epithelial sacs found in the larvae, have been popular tissues for tumorigenesis studies in *Drosophila* (Elsum et al., 2012; Herranz et al., 2016; Mirzoyan et al., 2019). During metamorphosis, these discs undergo major morphogenetic changes precipitated by intrinsic hormonal pathways and transform into exoskeletal structures including the head, thorax, and appendages (Blair, 2009). In total, there are 19 discs, each of which is formed from precursor cells that originate in the ectoderm of the embryo. Disc cells have a high rate of proliferation in the wondering larval stage, and the largest disc the wing disc can grow to a final size of approximately 50,000 cells (Aldaz & Escudero, 2010; Johnston et al., 1999).

The disc epithelial cells exhibit the archetypal apical-basal polarity. Their growth and proliferation are regulated by conserved signaling pathways. Deregulation of the growth signaling and disruption of cell polarity are two major causes leading to tumor formation in *Drosophila* epithelial tissues. The cell-polarity genes *scrib, lgl*, and *dlg* and their associated neoplastic growth phenotypes have been well characterized using the disc models (Herranz et al., 2016; Humbert et al., 2008). Another class of genes that may lead to neoplastic overgrowths when mutated in imaginal discs include regulators of vesicle and membrane trafficking, for example, *Tumor susceptibility gene 101 (TSG101), Vacuolar protein sorting 25 (Vps25)*, and *Rab5.* Not surprisingly, their mutants exhibit simultaneous cell-polarity loss and activation of growth-promoting signaling pathways (Gilbert et al., 2009; Herz et al., 2006; Thomas & Strutt, 2014). In this section, we focus mainly on recent developments using the imaginal-disc models to understand how different regulatory factors contribute to tumorigenesis in *Drosophila*.

The disc model has been used to determine the relationship between oncogenic mutations and tissue microenvironment during tumor initiation. It has long been known that oncogenic or tumor suppressor mutations do not always lead to neoplasms. For example, the *Rb* mutation, while commonly detected in many tissues, leads to tumorigenesis exclusively in the eye and bone (Ferres-Marco et al., 2006). With the advent of high throughput sequencing technology, cancer driving mutations have been found to be a lot more prevalent in healthy individuals than previously thought, raising questions about the exact role of these oncogenic mutations in cancer initiation (Martincorena et al., 2018). In this regard, the *Drosophila* wing disc model elucidates the role of tissue microenvironment in determining whether cells bearing oncogenic mutations can develop into a tumor. Applying mosaic generating tools, loss of *lgl* or *scrib* in different regions of the wing disc resulted in different consequences on tumorigenesis. Tumors grow specifically in the hinge region but not the pouch region of the wing disc (Tamori et al., 2016). A key difference between these tissue microenvironments is that JAK/STAT signaling is more potent in the hinge region than in the pouch. Another difference is the organization of the epithelium, which translates to discrepancies in the extrusion direction of pro-tumor cells. These findings have led to the concept of tissue tumor hotspot that describes the role of microenvironment in tumorigenesis (Tamori & Deng, 2017).

Similar to the wound healing process in humans, tissue regeneration in *Drosophila* involves genes and pathways that also regulate cell proliferation and growth. One such pathway is the Salvador-Warts-Hippo axis (Grusche et al., 2011), which is crucial in organ size control (Zhao et al., 2011). Yorkie (Yki), the *Drosophila* version of Yes-associated ptorin 1 (Yap), is a key transcriptional co-activator that serves a pivtal role in this axis (Sun & Irvine, 2011). By receiving upstream signals from atypical cadherin (Daschsoous and Fat) and myosin, Yki becomes the rate-limiting activator in *Drosophila* tissue regeneration. Yki is activated by wing disc damages via the Jun N-terminal kinase (JNK) pathway (Sun & Irvine, 2011). Thus, it is not surprising that a disregulated Yki can result in massive compensatory proliferation and organomegaly. It turns out that certain tumors can take advantage of this pathway during their dysplastic changes (Katsukawa et al., 2018). These studies help define the relationship between tissue regeneration and tumorigenesis.

The disc model has also been used to determine how genes involved in the epidermal–mesenchymal transition (EMT) promote tumor formation, similar to their human counterparts (Moreno-Bueno et al., 2008). *snail* (*sna*) and *serpent* (*srp*) are two EMT inducers in *Drosophila. srp* overexpression in wing discs leads to neoplastic transformation. The derived tumor grows many times larger after transplantation into a new host fly, and can continue to grow for many generations following repeated transplantation (Campbell et al., 2018). The underlying mechanism of *srp*-overexpression induced tumorigenesis involves activation of both Yki and Ras signaling. In contrast, overexpression of *sna* does not induce neoplastic growth, because *sna* activates only Yki but not Ras in this tissue (Campbell et al., 2018), suggesting that neoplastic growth involves multifaceted integration of signaling factors.

Epigenetic regulation is crucial in developmental plasticity and has been shown increased importance in tumorigenesis (Gonda & Ramsay, 2015). Most epigenetic regulators are evolutionarily conserved and are commonly mutated in human cancers (Piunti & Shilatifard, 2016). In *Drosophila*, the Polycomb group (PcG) and the Trithorax group (TrxG) chromatin proteins are major epigenetic regulators that function complementarily to maintain the repressed and active gene expression states, respectively (Geisler & Paro, 2015). A mutation of *Polyhomeotic* (*Ph*), a conserved member of the Polycomb repressive complex 1 (PRC1) in eye-disc cells, results in neoplastic growth (Y. R. Jiang et al., 2018). Loss of *Ph* leads to activation of *knirps (kni)*, an orphan nuclear hormone receptor and embryonic transcription factor, and reprogramming of cellular identity toward an embryonic state. Overexpression of *kni* could also lead to tumor formation in the larval eye disc. Both loss of *Ph* and *kni* overexpression share features such as ectopic activation of the JAK/STAT pathway and a block of differentiation (Torres et al., 2018).

In addition, PcG-mediated histone modifications regulate chromatin conformation within the irradiation-responsive enhancer region (IRER), an intergenic sequence that mediates the expression of surrounding proapoptotic genes, *reaper, hid*, and *grim* following ionizing irradiation (Y. Zhang et al., 2008). *dMyc*-induced apoptosis or hyperplasia is dependent on the epigenetic status of IRER. In flies with IRER deleted or in a closed epigenetic status, dMyc-overexpression induced apoptosis is blocked, whereas hyperplastic growth takes place, suggesting the important role of the epigenetic status of IRER in cellular decisions following oncogene-induced stress (C. Zhang et al., 2015).

Epigenetic regulators also include chromatin remodelers such as the Switch/sucrose nonfermentable (SWI/SNF) chromatin-remodeling complex, including *brahma (brm) or Snf5-related 1 (Snr1).* The *brm* and *Snr1* homologs are frequently mutated in human cancers (Reisman et al., 2009). Knockdown of *brm or Snr1* along with simultaneous overexpression of Yki using *apterous-Gal4 (apGal4)* leads to tumor growth in the dorsal compartment of the wing disc. SWI/SNF performs functions similarly to those of the Hippo pathway in suppressing Yki activity, which can induce tumor-like growth (S. L. Song et al., 2017). In a separate study, *Snr1* was shown to prevent tumorigenesis by maintaining an endosomal trafficking-mediated signaling cascade (Xie et al., 2017). Removal of *Snr1* resulted in neoplastic overgrowth in imaginal discs, whereas the depletion of any other members of the SWI/SNF complex failed to induce similar phenotypes. These findings are consistent with the fact that *SMARCB1*, the *Snr1* homolog in humans, is the only gene of the SWI/SNF complex responsible for malignant rhabdoid tumors (MRTs). It appears that the aberrant regulation of multiple signaling pathways, including Notch, JNK, and JAK/STAT, is responsible for tumor progression upon *Snr1* depletion. The tumor suppressor function of *Snr1* is largely dependent on its subcellular localization to the cytoplasm, rather than to the nucleus (Xie et al., 2017). Because MRTs are not of epithelial origin, the modeling of these pediatric tumors in *Drosophila* imaginal discs suggests the versatility of this system in understanding basic functions of tumor suppressor genes in normal development and their potential effects on tumorigenesis.

#### 2.1.2 | Imaginal rings

The imaginal ring is another imaginal tissue precursory to adult organogenesis in insects. Unlike the imaginal discs that form the exoskeletal structures in the adult, the imaginal rings eventually develop into the adult gastrointestinal organs, including the foregut (Fuss et al., 2004), hindgut (Tian et al., 2016), and salivary glands (Haberman et al., 2003). Notch signaling is activated in all three imaginal rings from the middle embryonic stage to the early pupal stage, and positively controls imaginal-ring cell proliferation during the third larval instar (Haberman et al., 2003; S. A. Yang & Deng, 2018). Overexpression of an active form of Notch, the Notch intracellular domain (NICD), induces overproliferation in these imaginal rings, similar to the hyperplasia growth phenotype induced by NICD in many imaginal discs (Djiane et al., 2013; S. A. Yang & Deng, 2018).

Interestingly, continuous overexpression of NICD in the salivary gland imaginal ring triggered tumor formation (S. A. Yang et al., 2019). These tumors form at the posterior end of the imaginal ring, which borders the polyploid giant-salivary-gland cells. This boundary region resembles the so-called “transition zones” known for their susceptibility to metaplasia, a premalignant lesion in mammalian epithelial tissues (Mcnairn & Guasch, 2011). Notch hyperactivation is sufficient to induce tumorigenesis at the salivary gland transition zone, but not in other imaginal ring cells. The underlying mechanism for tumor induction in this specific location is the endogenous high-level of JAK/STAT signaling and Matrix Metalloprotease 1 (MMP1) activity, induced by the JNK pathway. These tumors grow steadily in the host abdomen after transplantation and can reach the size about 30-times of the original tumor. Some tumor cells disseminate from the transplanted tumor and migrate to the host ovarian muscle sheath, oviduct, gut, and Malpighian tubule (S. A. Yang et al., 2019). This tumor model offers an opportunity to explore the effect of tissue microenvironment on tumor initiation and how host—tumor interactions affect tumor growth and metastasis.

### 2.2 | The central nervous system

The *Drosophila* central nervous system (CNS) consists of neurons organized into lineages, each of which descending from a single neuroblast specialized from a progenitor cell (Spindler & Hartenstein, 2010). The first round of neuronal divisions is performed by about 100 primordial neuroblasts, derived from the ectodermal layer around embryonic stage 10 (Younossi-Hartenstein et al., 1996). The neuroblast undergoes asymmetric division to produce a daughter neuroblast and a ganglion mother cell (GMC); the latter then generates two individual neurons via an additional division (Urbach & Technau, 2003). Although all cells in a proneural cluster have the potential, only one cell in each cluster is designated to become a neuroblast in CNS.

Cell fate specifications in the neural cell lineage involve cell—cell signaling mediated by the Notch pathway. The neuroblast expresses high levels of the Notch ligand Delta, thus activating Notch in the surrounding cells, inhibiting their differentiation into neuroblasts. In the larval stage, the neuroblast undergoes stereotypic, self-renewing divisions to produce INPs (immature neural progenitors), which, upon maturation, undergo a few more rounds of asymmetric, self-renewing divisions to give rise to ganglion mother cells (GMCs) that subsequently generate postmitotic neurons or glia (Bello et al., 2008). The disruption of cell-polarity and Notch pathway genes can potentially lead to tumorigenesis in the larval brain (Froldi et al., 2019; Goh et al., 2013; Y. Song & Lu, 2011).

Unidirectional Notch signaling that involves direct cell—cell contact between signal-sending and signal-receiving cells is both necessary and sufficient to promote neuroblast self-renewal (Bowman et al., 2008). At each neuroblast division, differentiation-promoting determinants are asymmetrically segregated. For example, the Notch antagonist Numb shows high levels of expression in immature INPs to suppress Notch signaling. Thus, the Notch pathway effector *HES* (*hairy* and *E[spl]*) genes are highly expressed in neuroblasts but not in immature INPs (Zacharioudaki et al., 2012). *numb* mutant INPs fail to complete maturation but instead revert back into the neuroblast fate and result in tumorigenesis in the larval brain (H. Wang et al., 2006).

Hyperactivation of Notch induced by changes in different Notch regulatory mechanisms leads to immature INP dedifferentiation and tumorigenesis, that is, the overproliferation of neuroblasts. One such regulation is achieved by the super elongation complex (SEC) which directly interacts with the Notch transcription activation complex, including dCSL (*Drosophila* homolog of *CSL*, also named *Suppressor of Hairless (Su(H)).* dCSL interacts tightly with the SEC subunit *AF4/FMR2 family-3 (dAFF-3).* The inactivation of SEC leads to neuroblast loss, whereas its forced activation results in neural progenitor dedifferentiation and tumorigenesis. Overexpression of the SEC subunits such as *eleven-nineteen lysine-rich leukemia (dELL)* and *dAFF* induces the expression of *hairy* and *E(spl))* genes (*dHES*), leading to a progenitor-derived tumor. These larval brain tumors can be sectioned and transplanted into the abdomen of young female adult hosts (Figure 2). Notably, only the tumor cells co-expressing *dELL* and *dAFF* or *dELL, dAFF* and *dCSL* can grow into large tumor masses and metastasize to the eye and thorax after xenograft of the tumor tissue into the adult abdomen. In contrast, the larval brain tumor tissue derived from dCSL-overexpression alone fails to metastasize in the adult host abdomen (Liu et al., 2017).

The cell polarity genes *lgl* and *atypical Protein Kinase C (aPKC)* have also been investigated extensively for their roles in neuroblast tumorigenesis (Homem & Knoblich, 2012). This is mainly due to their involvement in neuroblast asymmetric cell division, which often gives rise to an identical neuroblast as well as a differentiated daughter cell. This process begins with polarity establishment within the neuroectoderm during the embryonic stage, which is accomplished by the Bazooka (Baz) protein. The apically localized Baz directs the assembly of multiple downstream scaffolding complexes, which then coordinate enzymatic activities involved in asymmetric divisions (Gómez-López et al., 2014). Next, the mitotic spindle is oriented around the cellular scaffolds by the mushroom body-defective (Mud) protein. (Izumi et al., 2006). The final step in asymmetric division is cell fate determination, an intricate process directed by the Baz/Par6/aPKC complex and dependent on actin and myosin functions (Kraut et al., 1996).

Disruption of normally precisely controlled aPKC activity in neuroblasts can trigger tumor formation in larval brain. The aPKC/Baz/Par6 complex phosphorylates Numb and Miranda (Mira) to exclude them from the apical cortex, establishing the basal localization of cell fate determinants (C. Y. Lee et al., 2006). In *lgl* null mutants, Numb and Mira localization is defective, which leads to neuroblast overproliferation due to dysfunctional spindle formation (Atwood & Prehoda, 2009). The *Drosophila* ortholog of *clustered mitochondria homolog (CLUH), clueless (clu)*, has been found to promote *lgl*-induced tumorigenesis in the larval brain through regulating aPKC activity (Goh et al., 2013). Clu preferentially binds to the aPKC/Bazooka/Par6 complex and stabilizes aPKC levels.

A *Drosophila* larval CNS tumor model induced by the *lethal (3) malignant brain tumor (l(3)mbt)* mutation revealed the role of germline genes on tumorigenesis (Janic et al., 2010). The germline genes such as *nanos (nos), vasa (vas), P-element induced wimpy testis (piwi)*, and *aubergine (aub)* are upregulated in these tumor cells to induce a soma-to-germline transformation, which then help the larval brain tumor cells undergo self-renewal and hyperproliferation. Inactivation of these germline genes successfully suppressed tumor growth. Furthermore, blocking *piwi, vas* or *aub* in combination with the *l(3)mbt* mutation inhibited the transplanted tumor growth. It has been shown in various human malignancies, including melanoma and several types of carcinomas, that germline genes including *Melanoma antigen-1 (MAGEA1), MAGE-A2, ADAM metallopeptidase domain 2 (ADAM2)*, and cancer/testis antigen 1B (CTAG1B) become aberrantly activated (Akers et al., 2010). These genes, suspected to contribute to oncogenic traits such as immortality, invasiveness, and hypomethylation (Simpson et al., 2005), are being pursued as biomarkers and as targets for therapeutic cancer vaccines (Old, 2008). The similarities between the fly model and human cancers in germline gene upregulation present a promising new direction for future *Drosophila* cancer research (Gonzalez, 2013).

### 2.3 | The adult intestinal system

The *Drosophila* adult intestine is comprised of an ectoderm-derived foregut and hindgut, and an endoderm-derived midgut. The adult midgut is the functional equivalent of the mammalian small intestine where food digestion and nutrient absorption occur. The midgut epithelium is monolayered and contains self-renewing intestinal stem cells (ISC), the only dividing cell type in the midgut. After asymmetric division, an ISC produces a renewed ISC and either an enteroblast (EB) or a pre-enteroendocrine cell. EBs originate from the mitotic cell cycle and differentiate into absorptive enterocytes (ECs). Pre-enteroendocrine cells differentiate into mature enteroendocrine cells (EEs) (Miguel-Aliaga et al., 2018). This feature of a single-layer epithelium with divisional capabilities limited to ISCs makes the adult midgut an ideal model to explore tumorigenesis from stem cells (Singh et al., 2019).

In normal development, ISC division and differentiation are regulated by a combination of signaling pathways (H. Jiang et al., 2016). Their deregulation could lead to ISC overproliferation or loss of differentiation, which are major contributors to tissue overgrowth and tumor formation in the midgut model. Loss of *Notch (N)* has been shown to block differentiation, thus inducing an accumulation of ISCs (Micchelli & Perrimon, 2006; Ohlstein & Spradling, 2006). Along with loss of *N*, stress-induced divisions and an autocrine EGFR ligand *Spitz* are independently required for tumor initiation and early tumor growth in the ISC model (Patel et al., 2015). The stress-dependent cytokines Upd2 and Upd3 are induced to propel tumor growth without secondary mutations. In addition, *APC* mutation-driven intestinal adenoma has been shown to compete with surrounding cells by inducing apoptosis in the latter (Suijkerbuijk et al., 2016). Competition between the tumor cells and the surrounding enterocytes is mediated by JNK and Yki. *APC*^*−/−*^ mutant cells upregulate both JNK signaling and Yorkie activity, which in turn promotes tumor growth. The ISC model can thus be used to explore the relationship between tumor and the host microenvironment.

The *Drosophila* intestine has been used to model colorectal cancer (CRC), a multifactorial neoplasm involving genetic, environmental, and lifestyle risk factors (Brenner et al., 2014; Rawla et al., 2019). In humans, 40% of all cases of CRC involve mutations in the *KRAS* gene, while 70% show loss of function of *APC*. The combination of *APC* loss and *KRAS* mutation have been reported to cause an increase in adenoma size, number, and invasiveness (Cordero & Sansom, 2012; Janssens et al., 2006). In *Drosophila*, the activation of Wg/Wnt by loss of *APC* or the activation of Ras signaling in the midgut promotes ISC proliferation and causes tissue hyperplasia (Biteau & Jasper, 2011; H. Jiang et al., 2011; W. C. Lee et al., 2009; Martorell et al., 2014; C. Wang et al., 2013). Importantly, the combination of *APC* loss and *Ras* activation in the ISC model generates tumors with hallmarks of the human CRC (Martorell et al., 2014; C. Wang et al., 2013). These *APC-Ras* intestinal tumors are normally confined to the midgut, but the expression of the epithelial—mesenchymal transition (EMT) master inducer *snail (sna)* leads to the dissemination of tumor cells and macrometastases, which show parallels to human metastases (Campbell et al., 2019). *sna* activates a partial EMT in tumor cells, which then undergo collective cell migration and seed polyclonal metastases in multiple distant locations, including the ovary, thorax, and head, providing an excellent model for exploring metastatic behavior.

The adult hindgut, which has also been used to model colon cancer, is composed of large cuboidal epithelial cells confined to an anterior narrow segment called the hindgut proliferation zone (HPZ). Within the HPZ, self-renewal of ISCs, as well as subsequent proliferation and differentiation, are controlled by Wingless and Hedgehog (Hh) (Takashima et al., 2008). In the hindgut model, introducing a combination of multiple genes, including *Ras*^*G12V*^ and knockdowns of *p53, APC, Pten,* and *dSmad4*, can recapitulate key multigenic features of human colon cancer (Bangi et al., 2016). These features include the proliferation, disruption of the epithelial architecture, evasion of apoptosis and senescence, epithelial—mesenchymal transition (EMT), migration and cell dissemination to distant sites. Utilizing this multigenic model, the *Drosophila* hindgut has been used as a platform to investigate drug—tumor interaction and to screen various antineoplastic medications.

### 2.4 | The reproductive tissue

#### 2.4.1 | Ovarian germline stem cells

Within the female reproductive system, cells that have been reported to undergo tumorigenesis include the germline stem cells (GSCs) and the somatic follicle cells in the adult ovary. The ovaries are made of ovarioles, tube-like structures consisting of developing egg chambers and the anteriorly located germarium, where the GSCs are located. Asymmetric division of the GSC generates a daughter stem cell that retains contact with the somatic cap cells. The posterior daughter cell, however, is dissociated from the cap cells to differentiate into a cystoblast, which continues to undergo incomplete cytokinesis to form a 16-cell cyst. One of the cyst cells further differentiates into an oocyte, whereas the remaining 15 cells become polyploid nurse cells. The egg chamber, the developmental unit of oogenesis, is formed as the somatically derived follicle cells migrate to wrap the 16-cell cyst in the germarium (Greenspan et al., 2015; Hudson & Cooley, 2014).

Ovarian germline tumors in *Drosophila* are usually formed when GSC differentiation is blocked and continued divisions occur. Bag-of-Marbles (Bam), a member of a deubiquitinase complex to stabilize Cyclin A, is a key differentiation-promoting protein for cell-fate switching from a self-renewing stem cell to a differentiation-competent cystoblast. Bam is normally expressed in cystoblasts. Premature expression of Bam leads to GSC loss, whereas its suppression results in continued divisions of the GSC, producing GSC tumors. Disruption of *bam*, which can be achieved by the loss of Bam interaction partners in the cystoblast, is responsible for the majority of GSC tumor phenotypes reported so far (Iovino et al., 2009; Ohlstein & McKearin, 1997). Benign gonial cell neoplasm (Bgcn), Sex-lethal (Sxl), Mei-P26, and Twin are all factors related to RNA processing of *Bam.* Individual loss of any one of these in the female germline gives rise to the GSC tumor (Chau et al., 2009; Fu et al., 2015; Li et al., 2013; Ohlstein et al., 2000).

Bam expression in the cystoblast requires effective cell—cell communications elicited by the bone morphogenetic proteins (BMPs) from the somatic cells in the germarium. The BMP ligands Decapentaplegic (Dpp) and Glass-bottom boat (Gbb) are secreted from the somatic cap cells and target the GSCs via receptors Thickveins (Tkv), Saxophone (Sax), and Punt. This ligand-receptor interaction leads to phosphorylation of Mothers against Dpp (Mad), and its translocation into the GSC nucleus, where it associates with the *bam* promoter to suppress its transcription (L. Luo et al., 2015). In regions where BMP signaling is absent, Bam is expressed to facilitate germline differentiation. Many mutations that result in ovarian germline tumor formation, for example *tkv, fused, smurf*, and *mir-184*, are directly or indirectly involved in regulating BMP signaling in the GSC. Fused is a serine/threonine kinase that forms a complex with Smurf to control the fate of GSCs by generating a gradient BMP response (Xia et al., 2010). Mutations of micro-RNA, *miR-184*, which inhibits translation of the Sax receptor in wild-type cystoblasts, can also lead to GSC tumor formation (Iovino et al., 2009).

Maintenance of the GSC homeostasis requires the cooperation of different somatic cell types in the stem cell niche. An interesting example on how disruption of interactions among different somatic cell groups in the germarium leads to germline tumors involves the histone lysine-specific demethylase 1 (*Lsd1*) null mutation (Eliazer et al., 2011; H. Zhang & Cai, 2020). *Lsd1* functions specifically within the escort cells to prevent adjacent cap cells from ectopically producing niche-specific BMPs. Loss of *Lsd1* results in increased BMP signaling, which represses *bam* expression in the germline and leads to GSC tumor formation. Chromatin immunoprecipitation coupled with massive parallel sequencing (ChIP-seq) revealed over 100 genomic loci that are potential Lsd1 binding sites, including *engrailed*, which encodes a homeobox transcription factor (Eliazer et al., 2014). Knockdown of *engrailed* alleviated the stem-cell-expansion phenotype observed in *Lsd1* mutants. Interestingly, Knockdown of other potential direct Lsd1 targets that are unlikely BMP signaling components, also partially suppresses the *Lsd1* mutant phenotype. These results suggest that epigenetic regulator Lsd1 restricts the number of GSC-like cells by targeting a diverse group of genes to control the interactions among different cell groups in the germarium (Eliazer et al., 2014).

Similarly, depletion of *histone H1* in escort cells also leads to abnormal BMP signaling in germline cells and tumorigenesis. Knockdown of H1 allows escort cells to acquire the ability similar to the cap cells to produce Dpp, resulting in upregulated BMP activity to decrease Bam expression in spectrosome-containing germline cells (SCCs). These over-proliferative SCCs are the result of failed GSC differentiation into cystoblasts, probably taking on an intermediate fate. Interestingly, transposon activity increased dramatically in these H1-depleted escort cells (Z. H. Yang et al., 2017). Not surprisingly, loss of *Piwi*, a transposon silencer in escort cells, also resulted in GSC-like tumors in ovaries throughout adult life. The underlying mechanism appears to be similar to H1 knockdown in escort cells. *Piwi*-loss leads to Dpp upregulation in escort cells, which upregulates BMP signaling in the germline cells and blocks the differentiation of the GSCs (Z. G. Jin et al., 2013). Together, these studies suggest that abnormal epigenetic regulation of signaling events at the stem cell niche could lead to stem-cell derived tumorigenesis.

The control of Bam expression is also subject to regulation by sex determination genes. Maintenance of the sexual identity of the female germline has been shown to be crucial in preventing germline tumor formation. Differentiation from the GSC to cystoblasts requires physical interaction between Bam and Sex lethal (Sxl), the master regulator in the sex determination cascade. Loss of *Sxl* leads to a global upregulation of testis genes and a GSC tumor phenotype (Chau et al., 2009; Shapiro-Kulnane et al., 2015). The upregulated testis gene, *PHD finger 7 (Phf7)*, is a male germline sexual identity gene and a key effector of the tumor-forming pathway. *Phf7* upregulation is both necessary and sufficient for GSC tumor formation. In the female germline tumors with a loss of Bam expression, Phf7 is also upregulated, suggesting the critical role of sexual identify maintenance in the GSC tumor formation (Shapiro-Kulnane et al., 2015). Another *Sxl* related GSC tumor model involves an allele of *sans fille (snf)*, which specifically eliminates the Sxl protein in germline cells. The majority of *snf* tumor GSCs show testis-enriched markers and exhibit an intermediate fate between the stem cells and their differentiated daughter cells (Chau et al., 2009). These tumor cells also contain spectrosomes, similar to the ones produced when histone H1 is depleted in escort cells, suggesting the generation of the GSC- or GSC-like tumor cells converge on failed differentiation and adoption of an intermediate fate.

#### 2.4.2 | Somatic follicular cells

Tumors can be formed in somatically derived follicular epithelial cells in the ovarian tissue. These cells maintain a classic apical-basal polarity similar to epithelial cells in other tissues. Mutations in genes that show disruption of epithelial polarity (e.g., *lgl, srib*, and *dlg*) or endocytic membrane trafficking (e.g., *Vps, tsg101*) result in formation of multilayers of follicle cells (Bilder et al., 2000b; Tanentzapf et al., 2000; Vaccari & Bilder, 2005). Interestingly, this tumor-like multilayering is most prominent at the two termini of the egg chamber. Not coincidently, JAK–STAT signaling is strong in this terminal region, similar to the “tumor hotspots” in the hinge region of the wing imaginal disc and at the transition zone of the salivary gland imaginal ring (Beccari et al., 2002; McGregor et al., 2002). The precise role of JAK–STAT signaling in tumor formation at the egg chamber termini, however, requires further clarification.

The Notch pathway plays crucial roles in follicle cell differentiation and growth during multiple stages of oogenesis (Klusza & Deng, 2011). The role of Notch in the follicle cells is paradoxically opposite to its role in imaginal tissues. Notch activation triggers a transition from the mitotic cycle to the endocycle in follicle cells (Deng et al., 2001; Lopez-Schier & St Johnston, 2001). Disruption of Notch signaling leads to continued cell division, suggesting a tumor suppressor role of Notch in the follicle cells. The involvement of Notch in tumorigenesis in different *Drosophila* tissues is thus complex, ranging from an oncogenic role in imaginal tissues and neuroblasts and a tumor suppressor role in the reproductive and intestinal tissues. The *Drosophila* model may be key to further elucidating the diverse roles of Notch in tumor formation and growth.

#### 2.4.3 | Testis stem cells

The *Drosophila* testis contains two stem-cell populations: germline stem cells (GSCs) and cyst stem cells (CySCs) (Cheng et al., 2008; Voog et al., 2008). Maintenance of the stem-cell state requires JAK/STAT signaling from the hub. *upd* overexpression in germline stem cells leads to a striking overproliferation of early-stage cells which identified as a mixture of undifferentiated germline and somatic cells by their morphology and DNA staining characteristics. The overproliferated germ cells lack Bam consistent with a GSC and/or gonialblast identity (Tulina & Matunis, 2001).

Another model involves restoring the *Cdc25* homolog String (Stg) in GSCs in aged testis, causing late-onset tumorigenesis. *Stg* is highly expressed in young GSCs and CySCs and is required for stem cell maintenance. Interestingly, the expression of *Stg* declines with age. Restoring *Stg* expression by using germline specific *nanos-Gal4* in GSCs in aged testis prevents the age-associated decline in GSC proliferation and CySC number, but also causes tumors that were filled with undifferentiated GSCs and CySC-like cells. The germ cell tumors in these flies appear to be a mixture of GSCs and spermatogonia, which are confirmed by the presence of spectrosomes/fusomes (Inaba et al., 2011). Thus, the male and female germline tumors share some commonalities in the presence of spectrosomes, failed GSC differentiation and disrupted Bam expression.

### 2.5 | Hematopoietic cells

Hematopoiesis in *Drosophila* occurs throughout the life cycle. Embryonic hematopoiesis occurs at the head mesoderm anlage, whereas larval hematopoietic cells arise from the lymph gland (Holz et al., 2003). In adults, four blood cell clusters, called hematopoietic hubs, have been suggested to be located at the dorsal side of the adult fly abdomen (Ghosh et al., 2015a). Hemocytes or *Drosophila* blood cells can be divided into three subtypes: plasmatocytes, crystal cells, and lamellocytes. Plasmatocytes and crystal cells are hemocytes that account for about 90–95% and 2–5% of the total population, respectively. Lamellocytes, in contrast, are not normally present in healthy larvae but are instead induced by parasitic infection, tissue damage or hematopoietic tumorigenesis (Ghosh et al., 2015b; Lebestky et al., 2000). *Drosophila* hematopoiesis is used as a model to elucidate the basic mechanisms of hematopoietic differentiation and homeostasis, and for the study of blood cancers such as myeloproliferative neoplasms (MPNs) and acute myeloid leukemia (AML) (Outa et al., 2020).

MPNs are characterized by clonal proliferation of one or more hematopoietic cell lineages (Tefferi & Vardiman, 2008). The *Drosophila* hematopoietic tumors also involve overproliferation of either the lamellocytes or crystal cells and formation of melanotic masses (Minakhina & Steward, 2006). The JAK/STAT pathway, because of its prominent role in cell proliferation and differentiation in the hematopoietic lineage, is the major pathway that has been manipulated in *Drosophila* hematopoietic tumor models. Genetic manipulations of other pathways such as p38 MAPK, Ras, Toll, and JNK could also lead to increases of lamellocytes and formation of melanotic tumors (Zettervall et al., 2004). In addition, transgenic flies misexpressing a fused human gene resulted from the NUP98-HOXA9 translocation, a genetic lesion that leads to AML, helped to reveal a conserved molecular mechanism underlying blood cell expansion (Baril et al., 2017).

Gain-of-function mutations of JAK—STAT pathway components in the larval lymph gland can lead to lamellocyte overproliferation and alteration of the proportion of different hemocyte populations. The core pathway factors in JAK/STAT signaling include Hopscotch (Hop), the JAK2 homolog, and Stat92E, the only *Drosophila* STAT. Misexpression of a hyperactive *STAT92E* (*Stat92*^*EΔNΔC*^) by a pan-tissue driver *actin-Gal4* leads to melanotic tumor formation in the third larval instar (Ekas et al., 2010). The gain-of-function *hop*^*Tumorous-lethal (Tum-l)*^ mutant, which bears a glycine to glutamic acid substitution at residue 341 (G341E) in the Hop kinase, exhibits leukemia-like characteristics in larvae; the hemocytes show significant overproliferation, with ~50% of the circulating hemocyte population composed of lamellocytes and subsequent melanotic tumor formation (H. Luo et al., 1995). When transgenic *hop^Tum-l^* was expressed using the pan-hemocyte driver *Hml-Gal4*, the third instar larvae show increased plasmatocyte- and lamellocyte-counts in the hemolymph. Using this model for a modifier screen, mutants in the Hippo pathway were found to strongly enhance the melanotic tumor phenotype. Downregulation of Hippo pathway effector *Yki* resulted in a reduction in the number of hemocytes, which in turn downsized the melanotic tumor (Anderson et al., 2017). This study offers another example on the cooperation of growth regulating pathways in *Drosophila* tumorigenesis.

The *hop^(Tum-l)^* gain-of-function mutation induces a feed-forward mechanism that involves the activation of p38 MAPK, which in turn activates the cytokine-like ligand Upd3 and its receptor Domeless of JAK/STAT signaling, leading to lymph-gland hypertrophy (Terriente-Felix et al., 2017). In this feed-forward loop, Upd3 upregulation causes the overproliferation of Peroxidasin positive lamellocytes. Forced activation of the p38 MAPK pathway in maturing hemocytes is sufficient in generating hypertrophic lymph gland and melanotic tumors (Terriente-Felix et al., 2017). Because of the involved pathways are conserved, this pro-tumorigenic crosstalk between p38 MAPK and JAK/STAT signaling revealed in the *Drosophila* hematopoietic model is probably relevant in understanding the molecular mechanisms of human MPNs.

The Toll pathway plays a major role in innate immunity and is involved in hematopoiesis in *Drosophila* (Evans et al., 2003). Enhanced Toll signaling leads to an increase of lamellocytes appearing in circulation during the second instar and their abundance in the hemolymph of third instar larvae. Overexpression of a constitutively active Toll receptor *Toll^10b^* leads to an expansion of plasmatocyte and lamellocyte populations and a subsequent melanotic tumor formation. Loss of the IκB homolog *cactus*, a negative regulator of Toll signaling, leads to nuclear translocation of transcription factors Rel/NF-κB and differentiation of plasmatocytes into adhesive lamellocytes, followed by melanotic tumor formation. However, the factors downstream of the Toll pathway that regulate hemocyte division and differentiation remain unclear (Arefin et al., 2017; Qiu et al., 1998).

Epigenetic regulators are also involved in hematopoietic tumor formation in *Drosophila* larvae. PcG genes have been shown to act in conjunction with TrxG genes to maintain transcriptional regulation by altering chromatin status. The *PcG* gene *multi sex combs (mxc)* controls the proliferation and differentiation of the plasmatocyte lineage in both lymph glands and circulating hemocytes. Mutant alleles of *mxc* induce hematopoietic hyperplasia cell-autonomously. *brahma (brm)*, a TrxG gene and a SWI/SNF component, appears to be important for hematopoiesis during normal development and in *mxc* mutants. Furthermore, *Toll* is genetically epistatic to *mxc* in hematopoietic tumor formation. The interesting phenotype of the *mxc* hyperplastic lymph glands is their capability of transplanted growth for several generations (Remillieux-Leschelle et al., 2002).

The hematopoietic model offers a simple system to elucidate the molecular mechanisms underlying oncogenic *Ras* induced neoplastic growth (Asha et al., 2003). Expressing an activated form of Ras, Ras^V12^ by using the pan-hemocyte *Cg-Gal4* leads to 40-fold increase in the number of hemocytes, which express a pan-hemocyte antigen H2. The overproliferation of hemocytes also generates more functional plasmatocytes, which are capable of phagocytosis. Ras^V12^ induced hemocyte overproliferation is mediated by the Raf-MAPK pathway. Reduced activity of the *rolled (rl)* MAP kinase rescued the phenotype. Interestingly, the proliferative hemocytes induced by Ras^V12^ are able to divide continuously in the abdomen of the adult host, resulting in the death of 64% of the injected flies within 3 days (Asha et al., 2003).

The *Drosophila* hematopoietic tissue can be used to express human oncogenic or abnormal proteins to model AML, one of the most prevalent and lethal forms of leukemia (Baril et al., 2017). Homeobox (HOX) genes and cofactors are key regulators of hematopoiesis and their oncogenic potential was demonstrated by their ability to induce leukemia in distinct mammalian systems (Alharbi et al., 2013). A genetic lesion that involves the translocation and fusion of HOXA9 and NUP98 is a cause of AML. To reveal the underlying mechanism of *NUP98-HOXA9 (NA9)* in inducing leukemia, a *NA9* transgene was generated and overexpressed in the larval lymph gland with *Hml-Gal4*, which resulted in lymph-gland overgrowth with shared similarities with mammalian models (Calvo et al., 2002; Forrester et al., 2011; Takeda et al., 2006). NA9 influences signaling elicited by PVR, a PDGF/VEGF-like RTK related to several mammalian RTKs involved in leukemia (Baril et al., 2017). This model offers the possibility to tackle the underlying molecular mechanism of AML by genetic manipulations in *Drosophila*.

## 3 | *DROSOPHILA* MODELS FOR TUMOR–HOST INTERACTIONS

Current research on tumor—host interactions in the fly model focus on two areas, the innate immune response to tumor growth and the cachexia phenotype exhibited by the tumor host. The immune system is a double-edged sword in cancer progression. The host immune response that is meant to inhibit tumor growth may also end up promoting it. In an imaginal-disc tumor model generated by a loss of *scrib* and activation of Ras signaling (*Ras^V12^, scrib^−/−^*), the number of circulating hemocytes are significantly upregulated and some are attached to tumor cells via migration through the basement membrane. However, the effect of this association is controversial. In one study, hemocyte-induced apoptosis of *scrib* mutant imaginal discs cells inhibited tumor growth (Pastor-Pareja et al., 2008). In another study, the hemocytes were shown to bind to the tumor cells and secrete *eiger*, which then activated the TNF pathway to promote the growth of *Ras^V12^, scrib^−/−^* tumors (Cordero et al., 2010). This discrepancy indicates the complex role of hemocytes in tumor growth, which may be affected by other environmental and physiological conditions.

The fat body, which performs functions similar to the mammalian adipose tissue and liver also exhibits innate immunity function. Recently, a report showed that ectopic expression of antimicrobial peptides (AMPs), small cationic molecules that act in innate defense against microbial infection, in the fat body significantly suppressed the *mxcmbn1* lymph-gland hyperplasia phenotype. Two AMPs, Drosomycin and Defensin, are also produced by circulating hemocytes to inhibit tumor growth (Araki et al., 2019). Another report showed that Defensin is derived from the tracheal and fat body, and it binds to tumor cells in phosphatidylserine-enriched areas and promotes tumor-cell death and tumor regression (Parvy et al., 2019). The macrophage-like immune response to *Drosophila* tumors could provide useful information for immunotherapy research.

A major host response leading to health decline and mortality among cancer patients is cachexia, a systemic wasting syndrome with which the patient shows significant weight loss, especially as a result of skeletal-muscle atrophy and adipose loss. About 40–80% of cancer patients suffer cachexia, which accounts for about 20% of cancer deaths (Argiles et al., 2014). So far, no biomarkers or treatments have been discovered to be specific for cachexia. The initiators and the mechanisms underlying cachexia are currently unknown. Using the *Drosophila* tumor model to explore cachexia, Kwon et al. (2015) found that ImpL2, an IGF (insulin-like Growth Factor)-binding protein (IGFBP), is highly expressed in Yki-dependent tumor intestinal cells. The ImpL2 is sufficient in mediating the cachexia phenotype, which includes the wasting of muscle, fat body, and ovaries (Kwon et al., 2015). In another model with transplanted *scrib, Ras^G12V^* imaginal discs, ImpL2, the same cachexia regulator, is sufficient in inducing tissue wasting. Interestingly, only malignant tumors but not overproliferated tissue can cause cachexia (Figueroa-Clarevega & Bilder, 2015). These results illuminate new directions of exploration. For example, the maintenance of insulin balance can be important in the prevention of cachexia-like wasting. Currently, an increasing number of *Drosophila* tumor models have been applied to explore the cachexia-like wasting tumor—host interaction, which can be applied for drug screening to find cures for this devastating disorder.

## 4 | CONCLUSION

*Drosophila* has a long history of solving complex problems with simple but powerful genetic tools. This review details recently established tumor models using various *Drosophila* tissues from larval and adult stages, focusing on the tumor initiation process and the application of these tumor models in advanced research. When we compare these tumor models, several common features and key differences arise. First, *Drosophila* stem cells are highly prone to tumorigenesis, including neuroblasts, ISCs, GSCs, and stem-cell-like hematopoietic cells. These stem-cell tumors develop when a mutation leads to failed differentiation and continued stem cell division. Markedly, the genes and pathways involved in stem-cell maintenance and renewal, as well as the organization of the stem-cell niches, appear to be tissue specific. In the GSC lineage, the regulation of Bam expression is crucial. In neuroblasts and adult midguts, Notch signaling plays essential, albeit paradoxical roles, in stem cell maintenance. Second, the neoplastic tumor suppressor genes involved in cell polarity (*lgl, dlg*, and *scrib*) can induce tumor growth in multiple larval and adult tissues. These include the imaginal discs, neuroblasts, follicle cells, hemocytes and ISCs. Third, the activation of inflammatory signaling pathways including the JNK and JAK/STAT pathways appears to be quite common in tumor initiating microenvironments. Tumorigenesis in *Drosophila* also commonly involves cooperation of multiple growth regulatory pathways and epigenetic modifiers. The disruption of these pathways in specific cell types and tissue locations can lead to sustained cell proliferation and evasion of growth suppression. Some tumor types will gain replicative immortality after transplantation into a host fly and become metastatic. Lastly, tissue-specific malignancies could differ in response to the same oncogenic stimuli. For example, although the overexpression of NICD led to hyperplasia in many tissues, only the transition zone cells in the salivary gland imaginal ring undergo neoplastic growth (Gong et al., 2021; Yang et al., 2019). Further studies are needed to explore such tissue-specific responses to oncogenic stimuli. This can help researchers understand why certain regions are more “cancer-prone” than others. With the introduction of new sequencing and single cell technologies, the *Drosophila* tumor models are poised to contribute even more during the coming years to address questions in emerging fields in cancer research such as the tumor microenvironment and tumor—host interactions.

## Sidebar: Tumor transplantation (xenograft) in *Drosophila*

In the mammalian model, the most common strategy used in cancer transplantation research is injecting tumor cells into the abdominal wall or caudal vein of nude mice. The injected neoplastic cells could be of human, rat, or mouse origin. Human cancer cells could be derived from stable cancer cell lines or freshly prepared tissues from cancer patients. These cells can form tumors within the abdominal wall or in different organs of the recipient mice (Céspedes et al., 2007; K. Jin et al., 2010; O’Brien et al., 2007). Similarly, *Drosophila* tumors can be incised, and injected into the abdomen of a host fly. Depending on their genetic makeup, some of the transplanted tumors can grow much larger than the original tumor and eventually killing the host after a period of incubation. The original tumor can be induced in larval or adult stages by the disrupting a single or a combination of a cancer-related genes, as shown in Figure 2 (Figueroa-Clarevega & Bilder, 2015; Y. R. Jiang et al., 2018; S. A. Yang et al., 2019). Using the transplantation models, the tumor—host interaction can be explored.
